# RAS Nanoclusters Selectively Sort Distinct Lipid Headgroups and Acyl Chains

**DOI:** 10.3389/fmolb.2021.686338

**Published:** 2021-06-17

**Authors:** Yong Zhou, Alemayehu A. Gorfe, John F. Hancock

**Affiliations:** Department of Integrative Biology and Pharmacology, University of Texas Health Science Center, Houston, TX, United States

**Keywords:** RAS nanoclusters, phospholipids, electron microscopy, mitogen-activated protein kinases, depolarization, membrane curvature, cholesterol, polybasic domain

## Abstract

RAS proteins are lipid-anchored small GTPases that switch between the GTP-bound active and GDP-bound inactive states. RAS isoforms, including HRAS, NRAS and splice variants KRAS4A and KRAS4B, are some of the most frequently mutated proteins in cancer. In particular, constitutively active mutants of KRAS comprise ∼80% of all RAS oncogenic mutations and are found in 98% of pancreatic, 45% of colorectal and 31% of lung tumors. Plasma membrane (PM) is the primary location of RAS signaling in biology and pathology. Thus, a better understanding of how RAS proteins localize to and distribute on the PM is critical to better comprehend RAS biology and to develop new strategies to treat RAS pathology. In this review, we discuss recent findings on how RAS proteins sort lipids as they undergo macromolecular assembly on the PM. We also discuss how RAS/lipid nanoclusters serve as signaling platforms for the efficient recruitment of effectors and signal transduction, and how perturbing the PM biophysical properties affect the spatial distribution of RAS isoforms and their functions.

## Introduction

RAS isoforms, including HRAS, NRAS and KRAS4B are molecular switches that toggle between guanosine-5′-triphosphate (GTP)-bound active and guanosine diphosphate (GDP)-bound inactive states (Downward, 2003; Hancock, 2003; Cox et al., 2014; Cox et al., 2015; Prior et al., 2020). RAS proteins are key upstream regulators of the mitogen-activated protein kinases (MAPKs) signaling pathway, and participate in important cell functions including growth, division and proliferation (Cox et al., 2014; Cox et al., 2015; Prior et al., 2020). Mutations of RAS proteins are frequently found in many human diseases, and approximately 19% of all human cancers harbor RAS mutations (Cox et al., 2014; Cox et al., 2015; Prior et al., 2020). Mutations of KRAS4B are particularly prevalent in cancer, comprising ∼80% of all RAS-related oncogenic mutations (Downward, 2003; Hancock, 2003; Cox et al., 2014; Cox et al., 2015; Prior et al., 2020). Mutations of KRAS4B are found in 98% of pancreatic, 45% of colorectal and 31% of lung tumors (Downward, 2003; Hancock, 2003; Cox et al., 2014; Cox et al., 2015; Prior et al., 2020). Despite >30 years of intense research, KRAS remains difficult to directly inhibit by small molecule ligands (Ledford, 2015). Targeting the interactions of RAS with the plasma membrane is an attractive alternative because: 1) normal and aberrant biological functions of RAS proteins, including the constitutively active oncogenic RAS mutants, are mostly restricted to the plasma membrane (PM); 2) the distinct C-terminal membrane-anchoring domains of RAS isoforms contribute to their isoform-specific biological activities; 3) RAS dimerization occurs only on the PM and contributes to the formation of RAS signaling platforms on the PM. In this review, we will discuss the latest findings on how RAS isoforms undergo spatial distribution on the PM. We will specifically discuss the selective interactions of RAS proteins with distinct PM lipids, their lateral dynamics, and dimerization and oligomerization via specific interaction interfaces. We will also discuss our perspective on how RAS-RAS and RAS-lipid interactions might be targeted to inhibit aberrant RAS signaling.

### Isoform-Specific Intracellular Transport of RAS

**FIGURE 1:**
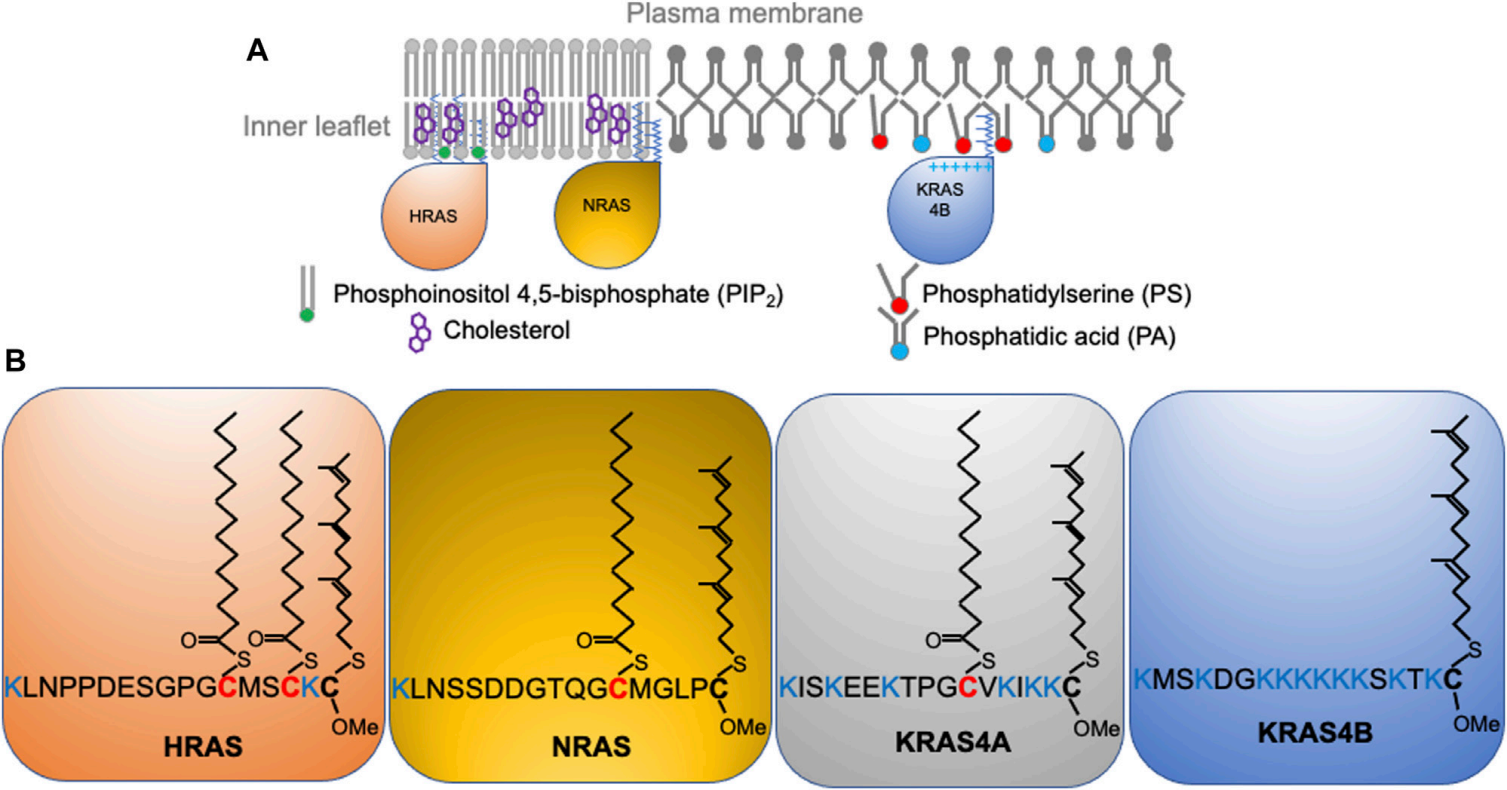
RAS isoforms with distinct C-terminal membrane-anchoring domains interact with different lipids and form spatially non-overlapping domains on the plasma membrane. **(A)** RAS proteins, including HRAS, NRAS, and KRAS4B distribute to distinct locations on the inner leaflet of the plasma membrane. **(B)** RAS isoforms share near identical enzymatic G-domains (>95% homology) and variable C-terminal hypervariable regions (HVR). RAS isoforms undergo distinct posttranslational modifications to add acyl chains to their HVRs for selective lipid sorting and nanoclustering.

Wild type RAS predominantly signals from the inner surface of the PM (Figure 1A) where recruitment and activation of effector proteins occurs (Hancock, 2003; Cox et al., 2015; Zhou and Hancock, 2015; Zhou and Hancock, 2017). This is also the case for the constitutively active oncogenic mutants of RAS. Thus, proper PM localization and spatial distribution of both wild-type and mutant RAS proteins is essential to biology and pathology. All RAS isoforms share nearly identical G-domains (>95% sequence identity) and highly divergent C-terminal hypervariable regions (<20% homology) (Figure 1B). All RAS isoforms undergo multiple steps of posttranslational modifications that add structural features required for membrane interaction, and are transported to the PM via various intracellular trafficking routes. First, farnesyltransferases recognize the C-terminal CAAX motif to irreversibly add a poly-unsaturated and branched 15-carbon farnesyl chain to the cysteine residue at position 185 (Reiss et al., 1990). The prenyl anchor allows RAS to localize to the cytosolic side of the endoplasmic reticulum (ER) membrane, where RAS converting enzyme (Rce1) cleaves the AAX residues of CAAX (Boyartchuk et al., 1997; Kim et al., 1999). The farnesylated Cys is then methyl-esterified at the α-carboxyl group by isoprenylcysteine carboxyl methyltransferases (ICMT) (Hrycyna et al., 1991; Dai et al., 1998). All RAS isoforms undergo these modifications, but diverge in their further processing. NRAS is palmitoylated at Cys181 and HRAS is palmitoylated at Cys181 and Cys184 (Figure 1B) by palmitoyltransferases at the Golgi apparatus (Hancock et al., 1991; Hancock et al., 1989) before being transported to the PM via the classic vesicular trafficking pathways (Hancock et al., 1991; Hancock et al., 1989). Palmitoylation is reversible, and the thioester bond in RAS palmitoyl cysteines can be cleaved by the PM-resident thioesterases (Ahearn et al., 2011). Depalmitoylated NRAS and HRAS fall off the PM and return to the Golgi apparatus and, following repalmitoylation, recycle back to the PM (Hancock et al., 1991; Hancock et al., 1989). The reversible palmitoylation/depalmitoylation cycle therefore dynamically regulates the intracellular trafficking of NRAS and HRAS (Hancock et al., 1991; Hancock et al., 1989). Other chaperons, such as VPS26A, VPS29, and VPS35 also facilitate the transport of NRAS between intracellular compartments and the PM. By contrast, KRAS4B is not palmitoylated but instead contains a polybasic domain (PBD) composed of six lysine residues (Lys 175-180) immediately before the site of farnesylation (Figure 1B). Unlike NRAS and HRAS, KRAS4B does not go to the Golgi apparatus (Hancock et al., 1991). Rather, the farnesylated KRAS4B molecules (Figure 1B) fall off the ER and undergo cytosolic diffusion facilitated by phosphodiesterase δ (PDEδ), which possesses a prenyl-binding pocket to sheath the farnesyl anchor of KRAS4B in the cytosol (Chandra et al., 2012; Schmick et al., 2015; Schmick et al., 2014). The fully processed KRAS4B, chaperoned by PDEδ, preferentially localizes to the recycling endosomes for delivery to the PM. It is still unclear how KRAS4B chooses the recycling endosomes, possibly facilitated by the electrostatic interactions between the KRAS4B PBD and anionic lipids enriched on the recycling endosomes (Chandra et al., 2012; Schmick et al., 2014; Schmick et al., 2015). Additionally, GPR31, a G protein-coupled receptor, also acts as a chaperon by associating with the farnesylated KRAS4B to aid in the transfer of KRAS4B to the PM (Fehrenbacher et al., 2017). Interestingly, intracellular transport of KRAS4B may not even need endomembrane organelles. A recent atomic force microscopy (AFM) study shows that KRAS4B can incorporate into membrane-less protein condensates formed by liquid-liquid phase separation (Li et al., 2021). The study revealed that the liquid droplets dissolve in the presence of a supported bilayer, with the released KRAS4B molecules attached to the bilayer and undergo nanoclustering (Li et al., 2021). Long thought a minor slice variant, KRAS4A is regaining attention in recent years with the discovery that it is widely expressed in many cancer cells (Tsai et al., 2015). KRAS4A is mainly localized to the PM but it also cycles among various endomembrane compartments. Its lipid anchor harbors two short segments of basic residues, a palmitoyl chain, and a farnesyl chain (Figure 1B), but unlike the similarly mono-palmitoylated NRAS, upon depalmitoylation KRAS4A localizes to the outer mitochondrial membrane (OMM) where it interacts with hexokinase 1 (Amendola et al., 2019). Taking together, the exiting data strongly suggest that the differences in the C-terminal membrane-anchoring domains of RAS isoforms contribute to their distinct intracellular trafficking properties.

### Isoform-Specific Nanoclustering of RAS

**FIGURE 2:**
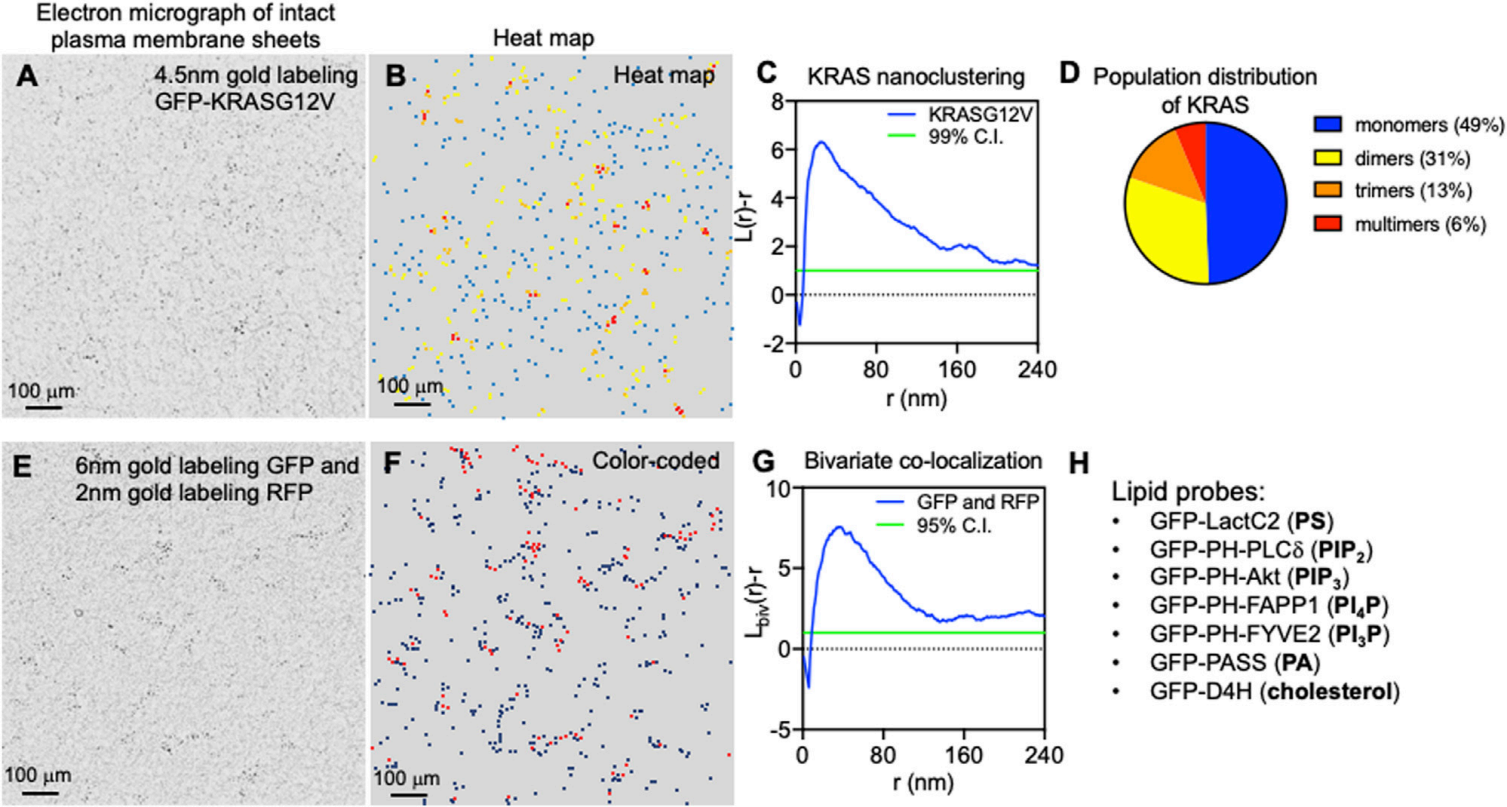
Super-resolution electron microscopy quantitatively characterizes the spatial distribution of RAS on intact plasma membrane sheets. **(A)** A sample electron micrograph of an intact plasma membrane sheet with an area of 1 μm^2^ is shown. Black dots indicate 4.5 nm gold nanoparticles conjugated to anti-GFP antibody that tag the GFP-tagged KRAS4B localized to the inner leaflet of the plasma membrane. **(B)** Gold nanoparticles are then color-coded to indicate spatial distribution in a heat map of the same electron micrograph as shown in *A*. **(C)** The Ripley’s K-function calculates the spatial distribution of the gold particles shown in *A* and *B*. Extent of nanoclustering, *L*(*r*)-*r*, is plotted as a function of radius *r* in nanometer. *L*(*r*)-*r* values above the 99% confidence interval (99% C.I.) indicate statistically significant nanoclustering. The peak *L*(*r*)-*r* value, termed *L*_*max*_, statistically summarizes the nanoclustering. **(D)** Further examination of the nanoclustering data in C allows calculation of the population distribution of cluster sizes. **(E)** A sample electron micrograph of an intact plasma membrane sheet with an area of 1 μm^2^ is shown. Two populations of gold nanoparticles are observed: 6 nm gold particles conjugated to anti-GFP antibody and 2 nm gold coupled to anti-RFP antibody. These gold particles are color-coded and shown in **(F)**. **(G)** The Ripley’s bivariate co-localization K-function calculates the co-clustering between the two populations of gold particles. Extent of co-clustering, *L*_*biv*_(*r*)-*r*, is plotted as a function of radius *r* in nanometer. *L*_*biv*_(*r*)-*r* values above the 95% confidence interval (95% C.I.) indicate statistically significant co-clustering. Integration of the *L*_*biv*_(*r*)-*r* curve between *r* values of 10 and 110 nm yields a statistical summary, termed as L-bivariate integrated (LBI), to indicate co-clustering. **(H)** Lists a cohort of specific lipid-binding domains used to probe the spatial distribution of some major lipids in the inner leaflet of the plasma membrane.

Once localized to the PM, RAS proteins undergo lateral segregation in the x-y plane to form nanometer-sized domains or nanoclusters, which serve as isoform-specific signaling platforms. In addition to RAS, these nanoclusters contain other proteins and lipids that are important for effector recruitment and signal propagation. Prior et al. was the first to quantify how immunogold-labeled RAS isoforms laterally distribute on intact PM sheets using electron microscopy (EM)-univariate nanoclustering analysis (Prior et al., 2003). In this analysis, intact PM sheets of mammalian cells expressing green fluorescence protein (GFP)-tagged RAS are attached to poly-L-lysine- and pioloform-coated copper (for apical PM) or gold (for basolateral PM) EM grids (Prior et al., 2003). The fixed intact PM sheets are labeled with 4.5 nm gold nanoparticles conjugated to anti-GFP antibody. Transmission electron microscopy (TEM) is used to image these gold-labeled PM sheets at a magnification of 100,000X. Figure 2A shows a raw EM image of an intact PM sheet of 1 μm^2^ area with gold-tagged GFP-KRAS4B. Figure 2B shows the same PM sheet, with the gold particles marked in different colors to illustrate the spatial distribution. ImageJ is used to assign the x, y coordinates for each gold particle. The Ripley’s K-function is then used to calculate the spatial distribution of these gold particles and to quantify the extent of nanoclustering of the gold-labeled GFP-RAS on intact PM sheets (Ripley, 1977; Diggle, 1979; Diggle et al., 2000). As shown in Figure 2C, the extent of nanoclustering, *L*(*r*)-*r*, can be plotted as a function of radius *r* in nanometer. *L*(*r*)-*r* values above the 99% confidence interval (99% C.I.) indicate statistically significant nanoclustering. The peak *L*(*r*)-*r* value, termed as *L*_*max*_, is generally used as a statistical summary for the nanoclustering event, which tightly correlates with the area-under-the-curve values of the K-function curve (Zhou et al., 2017). Number of neighboring gold particles within 15 nanometers of each gold is also calculated to estimate population distributions (Figure 2D). Other optical imaging techniques have been used to extensively validate the spatial distribution of RAS in intact and live cells. One of these is fluorescence lifetime imaging-fluorescence resonance energy transfer (FLIM-FRET), which has been used to measure the extent of co-localization of GFP- and RFP-tagged RAS in intact cells and tissues (Zhou et al., 2012; Zhou et al., 2014; Zhou et al., 2015; Zhou et al., 2017; Liang et al., 2019). The FRET efficiency between the GFP and RFP can be used to quantify close association (within 10 nm) among RAS molecules, and such measurements have been found to nicely correlate with the nanoclustering of RAS determined by the EM-spatial analysis. Raster image correlation spectroscopy (RICS), fluorescence correlation spectroscopy (FCS), fluorescence recovery after photobleaching (FRAP), total internal reflection fluorescence-single particle tracking (TIRF-SPT), and photoactivated localization microscopy (PALM), have also been used to measure the diffusion and population distribution of RAS monomers and nanoclusters in live cells (Murakoshi et al., 2004; Nan et al., 2015; Sarkar-Banerjee et al., 2017). Atomic force microscopy (AFM) has been used to image the lateral distribution purified full-length RAS proteins or the truncated minimal membrane anchoring domains on supported bilayers of co-existing lipid domains (Nicolini et al., 2006; Weise et al., 2011). *In silico* molecular dynamics (MD) simulations are also used to elucidate the physicochemical basis for the spatial segregation of RAS lipid anchors in one- or multi-component bilayers (Gorfe et al., 2004; Gorfe et al., 2007a; Gorfe et al., 2007b; Janosi and Gorfe, 2010; Janosi et al., 2012; Li et al., 2012). These quantitative super-resolution imaging and simulation studies consistently corroborate and demonstrate the spatiotemporal dynamics and isoform-specific organization of RAS proteins on membranes of different complexities.

As the sample Ripley’s K-function curve in Figure 2C illustrates, peak clustering of GFP-RAS occurs at the radial length r of ∼20 nm, suggesting that the most probable radius of GFP-RAS nanoclusters is approximately 20 nm (Prior et al., 2003; Plowman et al., 2005; Zhou et al., 2014; Zhou et al., 2015; Zhou et al., 2017; Liang and et al., 2019; Zhou et al., 2021). The K-function analysis further showed that RAS nanoclusters contain approximately 6–7 RAS molecules, and suggests that nearly half of GFP-RAS molecules exist as monomers, ∼30% as dimers, >10% as trimers, and <10% of GFP-RAS form higher order multimers (Zhou et al., 2017). This population distribution is consistent across a range of methods and data sources, including EM-spatial analysis of intact PM sheets, RICS and PALM analyses of live cell PM, as well as predictions from MD simulations (Janosi et al., 2012; Nan et al., 2015; Sarkar-Banerjee et al., 2017). Experiments using SPT, which tracks GFP-tagged RAS on the PM of live mammalian cells, found that the lifetime of RAS nanoclusters is between 100 ms and 1 s, with nanoclusters of the GTP-bound active RAS having a longer lifetime near 1 s (Murakoshi et al., 2004).

The local environment within different RAS nanoclusters is distinct since the nanoclusters are spatially segregated in an isoform- and guanine nucleotide-specific manner. This has been quantified using a special form of EM-spatial analysis, which is a bivariate co-clustering analysis using cells co-expressing GFP- and red fluorescence protein (RFP)-tagged proteins. In these experiments, EM is performed on intact PM sheets of mammalian cells co-expressing two different RAS isoforms (or the same RAS isoform bound with either GTP or GDP) tagged with GFP and RFP and co-labeled with 6 nm gold nanoparticles conjugated to an anti-GFP antibody and 2 nm gold nanoparticles coupled to an anti-RFP antibody (Prior et al., 2003). Figure 2E shows a raw EM image of an intact PM sheet of 1 μm^2^ area containing 6 nm gold tagging GFP and 2 nm gold tagging RFP, with the larger 6 nm gold marked in black and the smaller 2 nm gold marked in red (Figure 2F). After digitization via ImageJ, spatial co-clustering between the 6-nm gold and 2-nm gold particles is calculated via the Ripley’s bivariate co-clustering analysis. As illustrated in Figure 2G, extent of co-clustering, *L*_*biv*_(*r*)-*r*, is plotted as a function of *r* in nanometer. *L*_*biv*_(*r*)-*r* values above the 95% confidence interval (95% C.I.) indicate statistically significant co-clustering of the two populations of gold particles (Ripley, 1977; Diggle, 1979; Diggle et al., 2000). Such bivariate co-clustering analyses showed that co-clustering among HRAS, NRAS and KRAS4B is below the 95% C.I., suggesting minimal spatial overlap among the isoforms (Prior et al., 2003; Plowman et al., 2005). For each isoform, GTP- and GDP-bound RAS also show minimal co-clustering, indicating that the different nucleotide-bound forms of each RAS protein occupy distinct spaces on the PM inner leaflet (Prior et al., 2003). This spatial segregation is biologically important. For example, in a series of bivariate co-clustering analyses, acute depletion of cholesterol, via methyl β-cyclodextrin (MβCD), abolished the spatial segregation between the active GTP-bound HRAS and the inactive GDP-bound HRAS on the PM, and resulted in an inhibition of HRAS signaling (Ariotti et al., 2014). Elimination of caveolae on the PM, via knocking down important caveolar structural component caveolin 1 (CAV1), also induced mixing of the active GTP-bound and the inactive GDP-bound HRAS on the PM, which compromised HRAS signaling (Ariotti et al., 2014). Taken together, RAS proteins form lateral nanoclusters on the PM in isoform- and guanine nucleotide-specific manners.

### RAS Nanoclusters are Proteolipid Nano-Assemblies Acting as Signaling Scaffolds

RAS nanoclusters are the sites for effector recruitment and signaling (Hancock, 2003; Tian et al., 2007; Zhou and Hancock, 2015; Zhou and Hancock, 2017). They concentrate multiple RAS molecules within a small area of ∼300 nm^2^ on the PM (Prior et al., 2003; Plowman et al., 2005; Tian et al., 2007; Tian et al., 2010), increasing the probability of RAS-effector encounters. RAS nanoclusters are not exclusively “RAS oligomers” but rather molecular assemblies that contain other constituents needed for signaling propagation. The non-RAS constituents include lipids and other membrane-associated proteins, as well as the actin cytoskeleton structure. For example, EM-spatial analysis showed that nanoclustering of GFP-HRAS.GDP or GFP-KRAS4B.GTP was compromised upon Latrunculin A treatment to disrupt actin polymerization (Plowman et al., 2005; Zhou et al., 2014). Thus, actin is an important component in the nanoclustering of HRAS and KRAS4B on the PM. Expression of galectin-1 (Gal-1) enhanced the clustering of the constitutively active GFP-HRASG12V (Rotblat et al., 2004; Belanis et al., 2008), suggesting that Gal-1 is likely also a component of HRAS nanoclusters. This is supported by the observation that higher Gal-1 levels enhanced HRAS effector binding, MAPK signaling, and stemness of mutant HRAS-transformed mammalian cells (Blazevits et al., 2016; Posada et al., 2017). Furthermore, integration of molecular dynamics simulations, FLIM-FRET and EM-univariate nanoclustering analysis revealed that Gal-1 dimers formed complexes with the RAS-binding domain of RAS effectors, such as CRAF (Blazevits et al., 2016). This, in turn, stabilized nanoclusters of the GTP-bound active HRAS on the PM. Higher levels of galectin-3 (Gal-3), on the other hand, promoted the nanoclustering and effector binding of GFP-KRAS4BG12V (Elad-Sfadia et al., 2004; Shalom-Feuerstein et al., 2008), suggesting that Gal-3 is an integral component of the nanoclusters of active KRAS. Additional regulators of KRAS4B nanoclustering have been discovered through an extensive proteomic screen. These include nucleophosmin and nucleolin (Inder et al., 2009; Inder et al., 2010). Although primarily localized to the nucleus, a subset of nucleophosmin and nucleolin localize to the PM inner leaflet and become incorporated into KRAS4B nanoclusters, which results in further stabilization of KRAS4B nanoclusters and elevation of KRAS4B effector binding and MAPK signaling (Inder et al., 2009; Inder et al., 2010). FLIM-FRET and EM analysis showed that expression of the apoptosis-stimulating p53 protein (ASPP) family member, ASPP2, enhanced the nanoclustering and effector binding of HRASG12V, KRAS4BG12V and NRASG12V (Posada et al., 2016). Concordantly, expression of ASPP2 promoted MAPK signaling in mammalian cells transformed by HRASG12V, KRAS4BG12V or NRASG12V (Posada and et al., 2016). FLIM-FRET analysis and signaling assays revealed that ASPP2 competed with Gal-1 within the nanoclusters of HRASG12V and KRAS4BG12V (Posada et al., 2016). This competition resulted in an ASPP2-induced senescence of HRASG12V- and KRAS4BG12V-transformed mammalian cells, and abolished the HRAS- and KRAS4B-dependent formation of mammospheres of breast cancer cells (Posada and et al., 2016). Taken together, RAS nanoclusters on the PM are comprised of multiple protein and lipid constituents that, together, are important for effector recruitment and signal transduction.

### RAS Nanoclusters Sort Lipids in a Headgroup- and Acyl Chain Structure-Specific Manner

Lipids are the major constituents of RAS nanoclusters on the PM. These lipids are not only important for the structural integrity and stability of RAS nanoclusters, but also directly participate in effector recruitment. This is because most effectors of RAS contain specific lipid-binding domains and require synergistic association with both GTP-bound active RAS and a specific set of lipids for an efficient PM targeting and activation (Ghosh et al., 1994; Ghosh et al., 1996; Li et al., 2018). Even constitutively active mutants of RAS require precise spatial organization and lipid sorting to efficiently recruit their effectors and propagate signals (Inder et al., 2008; Inder and Hancock, 2008). For example, a major KRAS4B effector, CRAF, contains binding domains for both phosphatidylserine (PS) and phosphatidic acid (PA) (Ghosh et al., 1994; Ghosh et al., 1996; Li et al., 2018). It has been shown that the presence of PS and PA in membranes promoted the binding and activation of CRAF in synthetic liposomes and cells (Ghosh et al., 1994; Ghosh et al., 1996). Moreover, phosphoinositol-3 kinase (PI3K), a major effector of HRAS, specifically recognizes phosphoinositol 4,5-bisphosphate (PIP_2_) in the PM and converts it to phosphoinositol 3,4,5-trisphosphate (PIP_3_) (Hemmings and Restuccia, 2012). Thus, a key biological function of RAS nanoclusters appears to involve concentrating distinct lipids appropriate for each type of RAS isoform to recruit its specific effectors. This partially explains how RAS isoforms that share the same set of effectors differ in their affinity for different effectors, including the fact that KRAS4B preferentially recruits RAF while HRAS favors PI3K (Stokoe et al., 1994).

The enrichment of specific lipids within different RAS nanoclusters has been investigated using EM-bivariate co-clustering analysis of GFP-tagged lipid-binding domains that bind specific lipids (some examples listed in Figure 2H) and RFP-tagged RAS proteins on intact PM sheets (Zhou et al., 2014; Zhou et al., 2015; Zhou et al., 2017; Liang et al., 2019; Zhou et al., 2021). These experiments were complemented by FLIM-FRET in live cells expressing RFP-tagged RAS isoforms and spike-labeled TopFluor-tagged fluorescent lipids exogenously supplemented to these cells (Zhou et al., 2014; Zhou et al., 2015; Zhou et al., 2017; Liang et al., 2019; Zhou et al., 2021). The EM co-clustering analysis showed that RFP-KRAS4BG12V co-localized extensively with the PS probe GFP-LactC2 and the PA probe GFP-PASS, but not with the PIP_2_ probe GFP-PH-PLCδ, the PIP_3_ probe GFP-PH-Akt or the cholesterol probe GFP-D4H (Zhou et al., 2014; Zhou et al., 2015). On the other hand, RFP-tagged GDP-bound HRAS or its truncated minimal anchor (RFP-tH) were found to co-localize with probes of PIP_2_ and cholesterol (Zhou et al., 2014; Zhou et al., 2015). The difference in cholesterol association between KRAS4B and HRAS is consistent with earlier studies where acute cholesterol depletion by treatment of cells with methyl β-cyclodextrin (MβCD) effectively disrupted the nanoclustering and signaling of GFP-HRAS.GDP and GFP-tH but not GFP-KRAS4BG12V or GFP-tK (Prior et al., 2003; Plowman et al., 2005). Concordantly, the purified full-length KRAS4B and tK partitioned into the cholesterol-poor liquid-disordered (*L*_*d*_) domains of supported bilayers, as observed in atomic force microscopy (AFM) (Weise et al., 2011). MD simulations predicted that tH preferred to localize at the boundary between the cholesterol-enriched liquid-ordered (*L*_*o*_) and *L*_*d*_ domains (Janosi et al., 2012; Li et al., 2012; Li and Gorfe, 2013), consistent with experimental findings that cholesterol depletion disrupted the nanoclustering of tH in the cell PM. That RFP-KRAS4BG12V does not co-localize with PIP_2_ is surprising because the membrane-anchoring domain of KRAS4B is comprised of a hexa-lysine domain (Figure 1B) that is expected to interact with the PM primarily via electrostatics. Instead, the selective enrichment of the monovalent PS and PA over the multivalent PIP_2_ suggests a significant non-electrostatic contribution.

Additional insights into the lipid composition of RAS nanoclusters came from experiments in cells involving depleting and then adding back of specific lipids. In this regard, PS is of particular interest because KRAS co-localized extensively with a PS-binding domain in EM-bivariate co-localization analysis, as well as FLIM-FRET (Zhou et al., 2014). PS is the most abundant anionic phospholipid in mammalian cells, and is asymmetrically enriched in the PM inner leaflet. Mammalian cells typically contain two PS synthases (PSS): PSS1 that catalyzes the conversion of phosphatidylcholine (PC) to PS and PSS2 that converts phosphatidylethanolamine (PE) to PS (Lee et al., 2012). To manipulate PS content, PSS1 in Chinese hamster ovarian (CHO) cells was knocked down to generate a mutant line, termed as PSA3 cells (Lee et al., 2012). When grown in dialyzed fetal bovine serum (DFBS), PSA3 cells generate 35% less total PS and markedly lower PS levels in the PM inner leaflet (Lee et al., 2012; Zhou et al., 2014; Zhou et al., 2015; Zhou et al., 2017; Liang et al., 2019; Zhou et al., 2021). In the DFBS-treated PSA3 cells, supplementation of ethanolamine (Etn), which is a ligand upstream of PSS2, stimulates PSS2 and dose-dependently (0–10 μM for 72 h) elevates PS in the PM (Lee et al., 2012; Zhou et al., 2014; Zhou et al., 2015; Zhou et al., 2017; Liang and et al., 2019; Zhou et al., 2021). Then, different extracts of mouse brain lipids were acutely added back (1-hour incubation) to the PS-depleted PSA3 cells (Zhou et al., 2014; Zhou et al., 2015; Zhou et al., 2017; Liang et al., 2019; Zhou et al., 2021). EM-univariate nanoclustering analysis of these cells showed that PS depletion effectively disrupted the nanoclustering and PM localization of GFP-KRAS4BG12V as well as the GFP-tK but had no effect on GFP-HRAS (Zhou et al., 2014; Zhou et al., 2015). Supplementation of Etn (0–10 μM for 72 h) dose-dependently elevated the nanoclustering and PM localization of GFP-KRAS4BG12V but not GFP-HRASG12V (Zhou et al., 2014). In the PS-depleted PSA3 cells, acute addback of mouse brain extract of PS, but not extracts of other lipids tested (PIP_2_, PE, PC or cholesterol), recovered the nanoclustering and PM localization of GFP-KRAS4BG12V (Zhou et al., 2014; Cho et al., 2015). PS depletion disrupted the co-localization of GFP-KRAS4BG12V and RFP-tagged CRAF and thereby KRAS4B-dependent MAPK signaling, both of which were restored back to control levels upon the acute addback of PS but not any of the other lipids tested (Cho et al., 2015; Zhou et al., 2015; Zhou et al., 2017). Table 1 summarizes how different lipid types with distinct headgroups impact the spatiotemporal organization and effector recruitment of KRAS. Taken together, RAS nanoclusters have distinct lipid contents that contribute to selective effector recruitment and signal propagation.

**TABLE 1:** Nanoclusters of KRAS selectively enrich the mixed-chain PS species.

Lipid acute back	KRAS PM localization	KRAS nanoclustering	Lipids enriched in KRAS nanoclusters	KRAS recruitment of effector RAF
Brain PIP_2_ ^a^	Unaffected	Unaffected	No	Unaffected
Brain PC^a^	Unaffected	Unaffected	Not tested	Not tested
Brain PE^a^	Unaffected	Unaffected	Not tested	Not tested
Brain PS^a^ ^,^ ^b^ ^,^ ^c^	Enhanced	Enhanced	Yes	Enhanced
DSPS (di 18:0 PS)^c^ ^,^ ^d^ ^,^ ^e^	Unaffected	Unaffected	No	Not tested
DOPS (di 18:1 PS)^c^ ^,^ ^d^ ^,^ ^e^	Enhanced	Unaffected	No	Unaffected
DLPS (di 18:2 PS)^c^ ^,^ ^e^	Enhanced	Unaffected	No	Unaffected
POPS (16:0 / 18:1 PS)^c^ ^,^ ^d^ ^,^ ^e^	Enhanced	Enhanced	Yes	Enhanced
SOPS (18:0 / 18:1 PS)^c^ ^,^ ^e^	Enhanced	Enhanced	Yes	Not tested

^a^ Cho et al., 2016 Mol Cell Biol.

b Zhou et al., 2014 Mol Cell Biol

c Zhou et al., 2017 Cell.

d Liang et al., 2019 Life Sci Alliance.

e Zhou et al., 2021 Proc Natl Acad Sci U S A.

### KRAS4B Nanoclusters Concentrate Phosphatidylserine Species With Specific Acyl Chain Structures

As already noted, KRAS4B is targeted to the PM primarily via its C-terminal lipid anchor harboring a hexa-lysine segment (Lys175-180, Figure 2B). Therefore, it has long been thought that charge-charge interactions dominate the association of the KRAS4B polybasic domain (PBD) with the PS- and PIP_2_-enriched negatively charged PM inner leaflet. In this context, a surprising finding in the lipid mapping analysis described above was the suggestion that KRAS4B-PM interaction may involve more than just electrostatic complementarity, because KRAS4B nanoclusters were found to be selectively enriched with the monovalent PS but not the multivalent PIP_2_ lipids. To further test this, different exogenous PS species were acutely added back to the PS depleted PSA3 cells and the nanoclustering of GFP-KRAS4BG12V was quantified using EM (Zhou et al., 2017; Liang et al., 2019; Zhou et al., 2021). These synthetic PS species have the same charged headgroup and thus can be assumed to have the same electrostatic interactions with the PBD of KRAS4B. Their distinct acyl chain length and unsaturation level, however, can be expected to result in different packing characteristics that would result in different structural properties of membranes. While all exogenously added PS species effectively transported to the PM (validated via measuring labeling density of the PS probe GFP-LactC2) (Zhou et al., 2017), only the PS species with unsaturated acyl chains effectively recovered the PM localization of GFP-KRAS4BG12V, while the fully saturated di18:0 PS (DSPS) did not (Zhou et al., 2017; Zhou et al., 2021). Intriguingly, only the mixed-chain PS species, 16:0/18:1 PS (POPS) and 18:0/18:1 PS (SOPS), effectively recovered the nanoclustering of GFP-KRAS4BG12V (Zhou et al., 2017; Zhou et al., 2021); the symmetric PS species, including DSPS, di18:1 PS (DOPS), di18:2 PS (DLPS), had no effect on the nanoclustering of GFP-KRAS4BG12V (Zhou et al., 2017; Zhou et al., 2021). Effects of different PS species with distinct acyl chain structures on the spatiotemporal organization of KRAS are summarized in Table 1. These data suggested that KRAS4B has the ability to recognize PS acyl chains and thus the structure of the bilayer core. Recruitment of effectors by KRAS4B was also found to be dependent on PS acyl chain structure. This has been shown by EM-bivariate co-clustering analysis of intact PM sheets as well as by FLIM-FRET analysis in intact cells, demonstrating that recruitment of RFP-CRAF by GFP-KRAS4BG12V was abolished by PS depletion and was selectively recovered by acute addback of only POPS, but not the other PS species that have been tested (Zhou et al., 2017). EM-bivariate co-clustering analysis further showed that only acute addback of the mixed-chain PS species (POPS and SOPS) induced co-clustering of GFP-LactC2 (a PS-specific binding domain) and RFP-KRAS4BG12V (Zhou et al., 2017). In sum, it is clear that KRAS nanoclusters are selectively enriched with mixed-chain PS species, and that KRAS4B possesses an exquisite capability to selectively target PS headgroups and sort PS species based on their acyl chain structure.

### Nanoclusters Mediate Distinct Responses of RAS Isoforms to Perturbations of Plasma Membrane Biophysical Properties

The PM is not a homogeneous medium whose contents respond to perturbations in a similar manner. Rather, it is a highly heterogeneous and compartmentalized organelle (Simons and Ikonen, 1997; Simons and Toomre, 2000; Veatch and Keller, 2002; Baumgart et al., 2003; Veatch and Keller, 2003; Simons and Vaz, 2004; Veatch et al., 2007; Simons and Gerl, 2010) containing diverse nanometer-sized domains of different biophysical properties that respond to perturbations in distinct manners. Similarly, variations in the composition of nanoclusters of different Ras proteins suggest that RAS isoforms may responded to changing PM properties in distinct manners (summarized in Table 2). An important component of the PM is cholesterol, which plays key roles in the heterogeneity of the PM. In particular, cholesterol preferentially associates with saturated lipids and facilitates lipid phase separation into co-existing cholesterol-enriched *L*_*o*_ and cholesterol-poor *L*_*d*_ domains (Simons and Ikonen, 1997; Simons and Toomre, 2000; Veatch and Keller, 2002; Baumgart et al., 2003; Veatch and Keller, 2003; Simons and Vaz, 2004; Veatch et al., 2007; Simons and Gerl, 2010). EM-spatial analysis revealed that acute cholesterol depletion by MβCD treatment significantly disrupted the nanoclustering of GFP-tagged inactive HRAS (GDP-bound) or the minimal membrane-anchoring of HRAS (tH) (Prior et al., 2003). On the other hand, cholesterol depletion by MβCD treatment had no effect on the nanoclustering of active GTP-bound HRAS, GTP- or GDP-bound KRAS4B, or the minimal membrane-anchoring domain of KRAS4B (tK) (Prior et al., 2003; Zhou et al., 2021). Thus, nanoclusters of inactive GFP-HRAS.GDP and active GFP-NRAS.GTP are cholesterol-dependent while nanoclusters of active GFP-HRAS.GTP, GFP-KRAS4B (both active GTP-bound and inactive GDP-bound) and GFP-NRAS.GDP are independent of cholesterol. This is consistent with results from atomic force microscopy (AFM) experiments, where KRAS4B was located in the cholesterol-poor *L*_*d*_ domains of supported bilayers while the palmitoylated NRAS anchor was located along the domain boundaries between the *L*_*o*_ and *L*_*d*_ domains (Weise et al., 2009; Weise et al., 2011). While domain preferences of tH have not been tested experimentally on supported bilayers, MD simulations predicted that it localized at *L*_*o*_/*L*_*d*_ domain boundaries (Janosi et al., 2012; Li et al., 2012; Li and Gorfe, 2013). Cholesterol stabilizes domain boundaries and therefore tH nanoclusters. Thus, the spatial distribution of RAS proteins responds to cholesterol depletion in an isoform-specific manner.

**TABLE 2:** Nanoclusters of different RAS isoforms respond to membrane perturbations in distinct manners.

Membrane perturbations	KRAS4B.GDP (or tK)	KRAS4B.GTP	HRAS.GDP (or tH)	HRAS.GTP	NRAS.GDP (or tN)	NRAS.GTP
Cholesterol depletion^a^ ^,^ ^c^ ^,^ ^g^	Unaffected	Unaffected	Disrupted	Unaffected	Unaffected	Disrupted
Depolarization^d^ ^,^ ^g^	Enhanced	Enhanced	Unaffected	Unaffected	Not tested	Not tested
Curvature^f^ ^,^ ^g^						
Positive curvature	Disrupted	Disrupted	Enhanced	Enhanced	Not tested	Not tested
Negative curvature	Not tested	Unaffected	Disrupted	Not tested	Not tested	Not tested
Actin^b^ ^,^ ^f^	Not tested	Enhanced	Enhanced	Unaffected	Not tested	Not tested
Caveolae^h^	Disrupted	Disrupted	Enhanced	Enhanced	Not tested	Not tested

a Prior et al., 2003 J Cell Biol.

b Plowman et al., 2005 Proc Natl Acad Sci U S A.

c Roy et al., 2005 Mol Cell Biol.

d Zhou et al., 2015 Science.

e Zhou et al., 2017 Cell.

f Liang et al., 2019 Life Sci Alliance.

g Zhou et al., 2021 Proc Natl Acad Sci U S A.

h Ariotti et al., 2014 J Cell Biol.

Another important membrane property is curvature, which defines cell morphology and plays key roles in cell migration and intracellular trafficking (Baumgart et al., 2011; Bigay and Antonny, 2012; McMahon and Boucrot, 2015). Most membrane proteins that are known to sense or modulate membrane curvature, such as ion channels, receptors and Bin/Amphiphysin/Rvs (BAR) proteins, have a significant portion of their surface exposed to lipids. In contrast, a much smaller surface of monomeric RAS is directly exposed to lipids (Figure 1A), suggesting that membrane curvature sensing or modulation by Ras may involve cluster formation. Indeed, MD simulations of tH and full-length HRAS have shown a direct link between cluster formation, domain-segregation, and stabilization of membrane curvature (Janosi et al., 2012; Li et al., 2012; Prakash et al., 2012; Li and Gorfe, 2013; Li and Gorfe, 2013; Li and Gorfe, 2014; Li and Gorfe, 2014; Lin et al., 2015). Conversely, EM analysis has revealed that elevating PM curvature disrupted the nanoclustering and PM localization of GFP-KRAS4B but enhanced those of GFP-HRAS (consistent for both the full-length constitutively active mutants and the truncated membrane anchors) (Liang et al., 2019). This observation was made under multiple experimental conditions: 1) in intact PM sheets with curvature manipulated by the expression of different curvature-molding BAR domains; 2) in live cells grown over nanobars that induced quantifiable curvatures of the basolateral PM; 3) in isolated PM blebs with curvatures induced by exposure to hypo- and hypertonic buffers; and 4) in two-component synthetic liposomes of different sizes and curvatures (Liang and et al., 2019). In particular, depletion of endogenous PS in PSA3 cells grown in DFBS effectively abolished the ability of GFP-KRAS4B to respond to changing PM curvature (Liang and et al., 2019), suggesting that PS may mediate PM curvature sensing by KRAS4B. In the PS-depleted PSA3 cells, acute addback of only the mixed-chain POPS, but not the fully saturated DSPS and the mono-unsaturated DOPS, effectively restored the ability of GFP-KRAS4B to respond to changing PM curvature (Liang and et al., 2019). This was further supported by surface plasmon resonance (SPR) measurements using two-component synthetic liposomes, where binding of the purified full length KRAS4B to synthetic liposomes composed of the mixed-chain POPC/POPS (80/20) was enhanced as the vesicles became larger and less curved (Liang et al., 2019). On the other hand, KRAS4B binding was found to be independent of the size of vesicles composed of the mono-unsaturated DOPC/DOPS (80/20) lipids (Liang et al., 2019). A series of mouse embryonic fibroblast (MEF) mutant lines has been used to examine how RAS-dependent signaling responded to changing PM curvature. In these cell lines, all endogenous RAS isoforms have been knocked out and a specific KRAS mutant is expressed to generate RAS-less MEF lines (Drosten et al., 2010). Incubating RAS-less MEF expressing KRAS4BG12V in hypotonic buffers, which flattened the PM, significantly enhanced the KRAS4B-dependent MAPK signaling (Liang et al., 2019). On the other hand, in a RAS-less MEF line expressing a constitutively active RAS effector BRAFV600E mutant (with no RAS present), flattening of the PM via hypotonic buffers no longer affected MAPK signaling (Liang and et al., 2019). Taken together, the spatial distribution of RAS proteins responds to changing membrane curvature in an isoform-specific manner, with curvature sensing of KRAS4B being PS species-dependent.

Another important membrane property is electrostatics, more specifically transmembrane potential. It has long been known that transmembrane potential is associated with important intracellular signaling processes involved in cell growth and proliferation, and is correlated with cancer (Blackiston et al., 2009; Sundelacruz et al., 2009). Depolarization of the PM, as well as expression of depolarizing potassium channels, has been linked to elevated growth and proliferation and diminished apoptosis (Blackiston et al., 2009; Sundelacruz et al., 2009). However, the mechanism(s) behind this phenomenon has been poorly understood. A recent study using EM, FLIM-FRET, and FRAP showed that depolarizing the PM by increasing the extracellular potassium concentration or glutamate stimulation enhanced the nanoclustering of GFP-KRAS4BG12V and GFP-tK on the PM of non-polarized and polarized mammalian cells, as well as intact tissues of *Drosophila* brain (Zhou et al., 2015). PM depolarization also promoted nanoclustering PS and PIP_2_ but not PA and PIP_3_ lipids (Zhou et al., 2015). Nanoclustering and signaling of GFP-KRAS4BG12V did not respond to changing transmembrane potential in the PS-depleted PSA3 cells, but sensitivity was restored by Etn supplementation to increase endogenous PS levels (Zhou et al., 2015). In wild-type *Drosophila* embryos, depolarizing the PM similarly elevated signal output of the KRAS4B-dependent MAPK cascade whereas MAPK signaling was insensitive to PM depolarization in *Drosophila* embryos expressing an inactive mutant of a PS flippase, ATP8B (Zhou et al., 2015). Since ATP8B actively maintains an asymmetric distribution of PS in the PM inner leaflet (Paulusma et al., 2008; Ha et al., 2014), deactivation of ATP8B effectively depletes PS in the inner leaflet. Taken together, these studies demonstrated that PS mediates the spatial redistribution and altered signaling of KRAS4B in response to changes in the PM membrane potential.

### Mechanisms of Selective Lipid Sorting by RAS Proteins

RAS and other small GTPases use one or a few fatty acid chains with or without a PBD to target membranes. It is therefore intriguing that they would selectively sort lipid headgroup features and acyl chain structures, as do RAS proteins. It is becoming increasingly clear that this capability allows RAS proteins to respond to modulations of membrane biophysical properties in isoform- and guanine nucleotide-dependent manners. To systematically explore the molecular mechanisms underlying lipid sorting by RAS proteins, a series of studies have been conducted using EM-univariate and -bivariate analyses, FLIM-FRET, and MD simulations. Among the key observations of these studies was that the cholesterol dependence of the GDP-bound HRAS clustering is largely dictated by its palmitoyl chains at Cys181 and Cys184 (Roy et al., 2005). The nanoclustering of the constitutively active GFP-HRASG12V (with dual palmitoylation) did not respond to MβCD-induced cholesterol depletion (Roy et al., 2005), suggesting that GFP-HRASG12V does not co-localize with cholesterol. By contrast, MβCD-induced cholesterol depletion disrupted the nanoclustering of GFP-HRASG12V.C184S (HRAS mono-palmitoylated at Cys181) but not GFP-HRASG12V.C181S (mono-palmitoylated at Cys184) (Roy et al., 2005). This data suggests that the palmitoyl chain attached to Cys181 is key to driving the association of HRASG12V with cholesterol. This is, indeed, consistent with the finding that MβCD-induced cholesterol depletion effectively disrupted the nanoclustering GFP-NRASG12V, which is mono-palmitoylated on Cys181 (Prior et al., 2003), and predictions from free energy calculations that the second plamitoylation of HRAS was not required for high-affinity membrane binding but instead may modulate lateral dynamics (Gorfe and McCammon, 2008).

Although the C-terminal membrane-anchoring domain of HRAS plays important roles in membrane interactions, the catalytic G-domain may also contribute in some way. In earlier studies using MD simulations, it was found that the HRAS G-domain dynamically engaged the membrane in a nucleotide dependent manner (Abankwa et al., 2007; Abankwa et al., 2008; Abankwa et al., 2010). When GDP bound, the HRAS G-domain stayed away from the membrane while the HVR interacted with lipids and the palmitoyl chains fully inserted into the bilayer core. When GTP bound, the G-domain swinged up by almost 100 degrees to directly interacted with membrane lipids (Abankwa et al., 2007). As a result, a number of charged residues in switch I and II regions, including β2-β3 loop, helices α4 and α5, now extensively interacted with polar headgroups of lipids in the bilayer. This upward swing of the G-domain of HRAS caused its membrane-anchoring domain to move away from the membrane, which pulled the palmitoyl chains partially out of the bilayer (Abankwa et al., 2007). The resulting disorder in the palmitoyl chains was proposed to promote favorable interactions with the more disordered and thinner cholesterol-poor lipid domains (Gorfe et al., 2007a; Gorfe et al., 2007b; Abankwa et al., 2008; Abankwa et al., 2010). This was consistent with EM data showing that the nanoclustering of the constitutively active and GTP-bound GFP-HRASG12V was insensitive to cholesterol depletion by MβCD (Prior et al., 2003).

Inspired by a previous MD study that suggested the non-equivalency of the lysine residues of the PBD of the minimal membrane-anchoring domain (tK) of KRAS4B (Janosi and Gorfe, 2010), recent studies have focused on the nanoclustering of a cohort of PBD mutants in which each of the positively charged lysine residues was individually mutated to the neutral glutamine: GFP-KRAS4BG12V.K175Q, KRAS4BG12V.K176Q, KRAS4BG12V.K177Q, KRAS4BG12V.K178Q, KRAS4BG12V.K179Q, KRAS4BG12V.K180Q. Each of these mutants contains five lysine, and thus the six mutants have an identical total charge. It was found that KRASG12V.K177Q and KRASG12V.K178Q were remarkably weak in terms of both nanoclustering and PM binding compared with the other PBD mutants (Zhou et al., 2017). Further EM-bivariate co-clustering analysis revealed that these equally charged PBD mutants sorted distinct sets of lipids. In particular, nanoclusters of KRAS4BG12V.K177Q and KRAS4BG12V.K178Q were depleted of PS but enriched with PIP_2_, while the other PBD mutants still maintained extensive PS content in their nanoclusters. On the other hand, nanoclusters of KRAS4BG12V.K175Q and KRAS4BG12V.K179Q contained higher levels of PIP_3_. Nanoclusters of KRAS4BG12V.K178Q also contained significantly higher levels of PA. Another interesting PBD mutant involves the phosphorylation of Serine 181 via activation of protein kinase G (PKG) or the phosphomimetic mutant S181D of KRAS4B. EM-bivariate lipid mapping revealed that nanoclusters of the phosphorylated and S181D KRAS4B were depleted of PS but enriched with PIP_2_ and PIP_3_ (Zhou et al., 2017).

**FIGURE 3:**
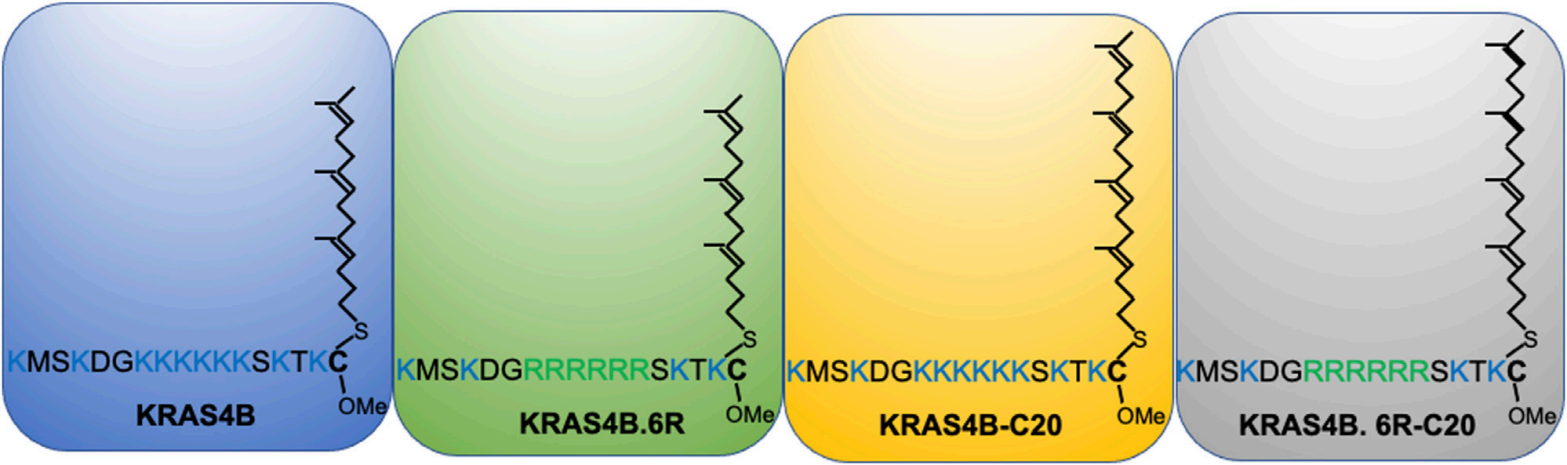
KRAS4B PBD mutants that share an identical number of positively charged residues and thought to electrostatically interact with the plasma membrane in an equivalent manner.

**FIGURE 4:**
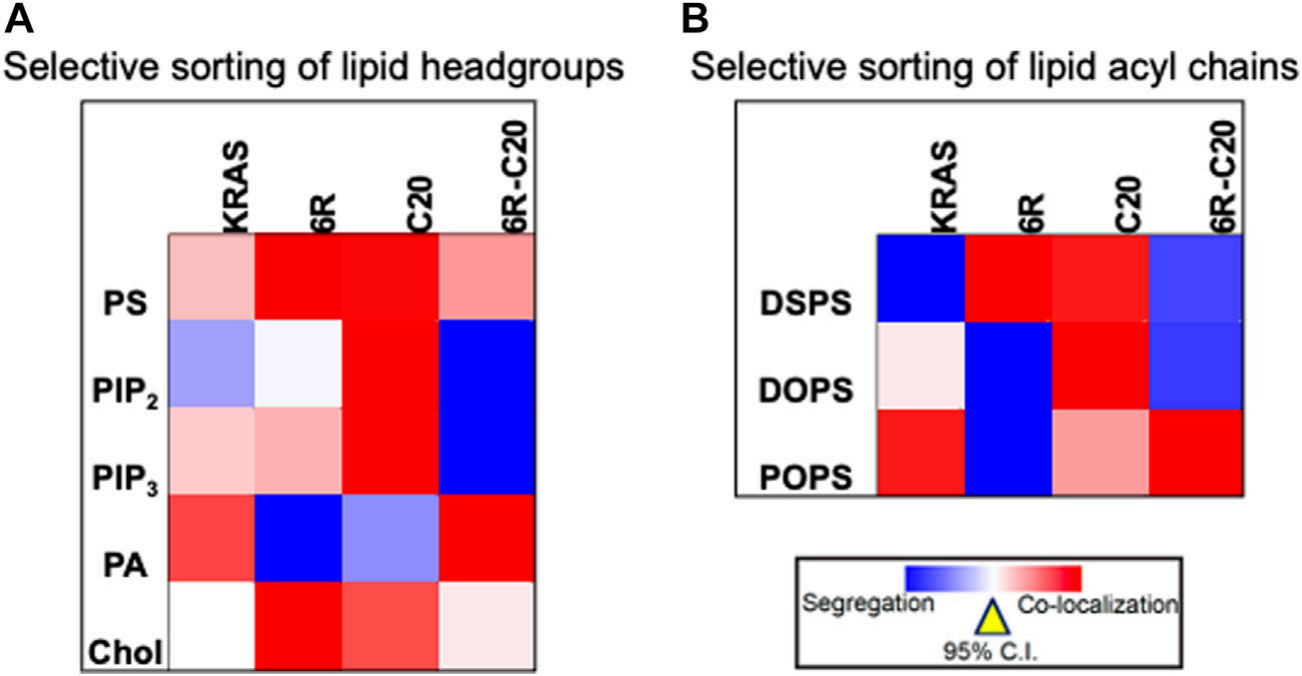
Equivalently charged KRAS4B PBD constructs selectively sort distinct lipid headgroups and acyl chains. **(A)** A heat map of LBI values indicates distinct co-clustering between the GFP-tagged specific lipid-binding domains and the RFP-tagged KRAS4B PBD constructs. **(B)** A heat map of LBI values indicates co-clustering between the PS-specific domain, GFP-LactC2 and the RFP-tagged KRAS4B PBD constructs in PS-depleted PSA3 cells following acute addback of distinct synthetic PS species.

Further evidence for the notion of not-just-electrostatics came from the comparison of four additional KRAS PBD constructs (Figure 3): GFP-KRAS4BG12V (with the original hexa-lysine PBD), GFP-KRAS4BG12V.6R (the six contiguous lysines replaced with arginines), GFP-KRAS4BG12V.C20 (the 15-carbon farnesyl chain mutated to the 20-carbon geranylgeranyl chain), GFP-KRAS4BG12V.6R-C20 (a geranylgeranylated hexa-arginine PBD). These four constructs contain an equivalent number of charged residues. However, while the nanoclusters of the reference KRAS4BG12V were enriched with PS as expected, those of KRAS4BG12V.6R and KRAS4BG12V.C20 became more enriched with cholesterol and depleted of PA while KRAS4BG12V.6R-C20 remained similar to the reference (Zhou et al., 2021) (data summarized in a heat map shown in Figure 4A). In addition to lipid headgroups, these equivalently charged KRAS4B PBD mutants also sort distinct lipid acyl chains. In acute lipid addback assays using PSA3 cells, it was found that the reference GFP-KRAS4BG12V co-localized extensively with the mixed-chain POPS but not the symmetric DSPS and DOPS (Zhou et al., 2021) (data summarized in a heat map shown in Figure 4B). On the other hand, GFP-KRAS4BG12V.6R co-localized with only the fully saturated DSPS while GFP-KRAS4BG12V.C20 co-localized with the symmetric DSPS and DOPS (Zhou et al., 2021) (Figure 4B). GFP-KRAS4BG12V.6R-C20 associated more preferentially with POPS (Zhou et al., 2021) (Figure 4B), again similar with KRAS4B with the original PBD. As a result, these KRAS4B with equivalently charged PBDs responded to changing PM properties in distinct manners. As summarized in Table 3, EM-nanoclustering analysis showed that, while GFP-KRAS4BG12V with the original PBD was independent of cholesterol, nanoclusters of GFP-tagged KRAS4BG12V.6R, KRAS4BG12V.C20 and KRAS4BG12V.6R-C20 were disrupted upon acute cholesterol depletion. The nanoclustering of GFP-KRAS4BG12V.6R also lost its sensitivity to PM depolarization (Table 3). Also interestingly, the nanoclustering of GFP-KRAS4BG12V.6R and GFP-KRAS4BG12V.C20 was enhanced by elevating PM curvature, opposite of the curvature preferences of the equivalently charged counterparts GFP-KRAS4BG12V and GFP-KRAS4BG12V.6R-C20 (Table 3). A mechanistic insight into how this might work at the atomic level emerged from atomistic MD simulations that predicted that the PBDs, including the original farnesylated hexa-lysine tK and mutants such as tK-K177Q and tK-K178Q sampled a large conformational space but differed in the proportion of ordered (O), intermediate (I) and disordered (D) backbone conformations (Zhou et al., 2017; Zhou et al., 2021). Approximately 64% of the simulated tK anchor was in the D state, 35% in the I (29%) and about 6% in the O state (Zhou et al., 2017; Zhou et al., 2021). These conformations differed in their capacity to form salt bridges involving the lysine side chains with the PS headgroups, with D state being the most amenable (Zhou et al., 2017; Zhou et al., 2021). Mutations that enriched the D state would therefore interact more favorably with PS lipids while those favoring the O state interacted less strongly. Consistent with this hypothesis and the experimental data described above, the less PS col-localizing tK-K177Q and tK-K178Q favored the O state (42 and 25% compared to 6% for tK) (Zhou et al., 2017). The geranylgeranylated tK, tK-C20, as well as the tK backbone phosphorylated at Serine 181, predominantly adopted the D states (Zhou et al., 2021). Taken together, the specific amino acid sequence and the prenyl anchor of KRAS4B together regulate the conformational plasticity of the prenylated PBD of KRAS4B and thereby determine its ability to selectively sort lipids.

**TABLE 3:** Nanoclusters of KRAS4B PBD constructs with identical numbers of charged residues respond to membrane perturbations in distinct manners.

Membrane perturbations	KRAS4B	KRAS4B.6R	KRAS4B-C20	KRAS4B.6R-C20
Cholesterol depletion^a^ ^,^ ^e^	Unaffected	Disrupted	Disrupted	Disrupted
Depolarization^c^ ^,^ ^e^	Enhanced	Unaffected	Enhanced	Enhanced
Curvature^d^ ^,^ ^e^				
Positive curvature	Disrupted	Enhanced	Enhanced	Disrupted
Negative curvature	Unaffected	Not tested	Not tested	Not tested
Actin^b^ ^,^ ^d^	Enhanced	Not tested	Not tested	Not tested
Caveolae^f^	Disrupted	Not tested	Not tested	Not tested

a Prior et al., 2003 J Cell Biol.

b Plowman et al., 2005 Proc Natl Acad Sci U S A.

c Zhou et al., 2015 Science.

d Liang et al., 2019 Life Sci Alliance.

e Zhou et al., 2021 Proc Natl Acad Sci U S A.

f Ariotti et al., 2014 J Cell Biol.

In addition to the PBD, the G-domain of KRAS4B may also contribute to lipid sorting. This is because the G-domain has been shown to dynamically interact with membrane lipids in at least two dominant orientational states (OS): OS1 and OS2. Helices α3 or α5 and α4 contacted the bilayer in OS1, whereas β1, β2 and β3 and helix α2 directly contacted the bilayer in OS2 (Mazhab-Jafari et al., 2015; Prakash et al., 2016; Sarkar-Banerjee et al., 2017; Li et al., 2018; Prakash et al., 2019; Prakash and Gorfe, 2019; Neale and García, 2020). As a result, each OS presented distinct polar residues to interact with charged lipid headgroups in membranes. For example, Arg97, Lys101 and Arg135 in OS1 and Arg73 and Arg102 in OS2 might interact with PS headgroups in the bilayer, respectively (Prakash et al., 2016). Additionally, the orientational states of the G-domain also impact the dynamics of the backbone of the polybasic region within the C-terminal membrane anchoring domain since the Lys175-180 segment was more extended in OS2 than OS1 (Prakash et al., 2016; Prakash and Gorfe, 2019). Moreover, the dynamic oscillation between OS1 and OS2 may contribute to lipid sorting of KRAS4B in ways that are yet to be elucidated. Along this line, EM analysis showed that mutating Arg73 to the oppositely charged Glu disrupted the nanoclustering of GFP-KRAS4BG12V.R73E on intact PM sheets (Prakash et al., 2016). Taken together, the orientational dynamics of the G-domain may complement the intrinsically disordered lipid anchor in the selective sorting of lipids by KRAS4B.

### RAS Dimerization Interfaces and Their Role in the Formation of High Order Oligomers

The PM provides a structural framework for both the signaling function and homodimerization of RAS proteins, and a growing body of evidence supports the notion that KRAS4B forms dimers and larger oligomers (or nanoclusters) in cells and synthetic membranes (Abankwa et al., 2007; Tian et al., 2007; Nan et al., 2015; Zhou and Hancock, 2015; Ambrogio et al., 2018). However, there are conflicting reports on whether oligomerization involves direct protein-protein interaction or is primarily mediated by lipids. Moreover, there are multiple predicted RAS dimerization interfaces [e.g., (Güldenhaupt et al., 2012; Muratcioglu et al., 2015; Sayyed-Ahmad et al., 2016)], which have been discussed in detail in several recent review articles included in refs (Abankwa and Gorfe, 2021; Van et al., 2021). There is debate regarding which of these interfaces is most relevant for function. We believe RAS utilizes various combinations of multiple interfaces to form oligomers of diverse sizes, topologies and internal structures. Such G-domain-mediated dimerization/oligomerization and lipid-mediated spatial segregation synergistically promote nanoclustering of RAS, which allows the formation of signaling platforms suitable for function in specific situations and pathways. With this in mind, here we will focus on two dimer models and how they might give rise to diverse high order oligomers.

Two partially overlapping protein-protein interaction interfaces (PPIs, termed i1 and i2) have been identified on the catalytic domain of KRAS4B by combining sequence analysis, protein-protein docking, and molecular simulations (Prakash et al., 2017). Potential of mean force (PMF) calculations suggested that both interfaces i1 and i2 were marginally stable in solution (calculated K_d_ ≈ 5 and 100 mM) (Prakash and et al., 2017). This was consistent with a previous report on the absence of KRAS4B dimers in solution (Werkmüller et al., 2013). However, MD simulations of the i1 and i2 dimer models attached to a POPC/POPS bilayer led to improved interactions, especially at interface i1, and stabilization of the dimers (Prakash et al., 2017). Using BHK cells ectopically expressing selected i1 mutants followed by biochemical assays and EM analysis, it was found that neither charge-reversal mutations of interfacial ion pairs (K101E and E107K) nor a charge-swapping double mutant (K101E/E107K) affected membrane targeting (Prakash et al., 2017). However, the charge-reversal, but not the charge swapping, mutation significantly reduced clustering relative to the wild type (Prakash et al., 2017). Introducing cysteines at the same positions (K101C/E107C) dramatically enhanced both membrane retention and clustering (Prakash et al., 2017), likely due to the formation of an intermolecular disulfide bond. Indeed, a corresponding QQ mutant that was unable to form a disulfide cross-link had no effect on membrane binding or clustering (Prakash et al., 2017). Moreover, by comparing dimer/monomer and oligomer/monomer ratios, it was found that the single-point charge reversal mutations reduced the dimer and higher oligomer fractions while the K101C/E107C mutation dramatically increased those fractions (Prakash et al., 2017). Further, immunoblotting the membrane fraction of wild type and K101C/E107C KRAS4B under a non-reducing condition indicated dimer and oligomer bands for both, with the latter being substantially more prominent (Prakash and., 2017). No oligomer bands were found in the cytosolic fraction (Prakash et al., 2017). A recent paramagnetic relaxation enhancement NMR spectroscopy revealed that GTP-bound active and GDP-bound inactive KRAS4B formed homodimers via an interface involving helices α4 and α5 (Lee et al., 2020). Specifically, electrostatic interactions between residue pairs of R135-E168, Q131-D154 and Q131-R161 contributed to the homodimerization of GTP-bound KRAS4B on bilayers, whereas dimers of GDP-bound KRAS4B was stabilized by E49-K172 and E162-K165 residue pairs. The α4/α5 interface of KRAS4B dimers has also been observed in size exclusion chromatography and small angle X-Ray Scattering (Packer et al., 2021). The presence of the RAS-binding domain of RAF further stabilized dimerization of KRAS4B on membrane. Combining FRET/electron paramagnetic resonance spectroscopy and MD simulations, a recent study also characterized helices α4 and α5 as an important dimer interface in NRAS (Rudack et al., 2021). Specifically, the most prevalent residue contact between the GDP-bound NRAS monomers was a salt bridge between D154 and R161 located on α5 (Rudack et al., 2021). Another prominent contact between the two NRAS monomers was between H131 of α4 helix and E49 of the β2-β3 loop (Rudack et al., 2021). These findings underscore the important role of helices α4 and α5 in stabilizing homodimers of RAS anchored to membranes. Taken together, these observations suggest that KRAS4B forms dimers and oligomers of diverse size and shape via interfaces i1 and i2 (Prakash and et al., 2017).

The above conclusion is further supported by a study that quantified the distribution of KRAS4B oligomers on the PM using a combination of single molecule experiments and molecular modeling (Sarkar-Banerjee et al., 2017). The study included fluorescence correlation spectroscopy (FCS) and FRAP in cells transiently expressing low levels of mGFP-tagged WT, K101E and K101C/E107C KRAS4B mutants (Sarkar-Banerjee et al., 2017). The FRAP analysis suggested K101E had a larger mobile fraction and a smaller percentage of cells with two distinct diffusivities. FCS showed that 50% (K101E), 58% (WT) and 89% (K101C/E107C) of the cells that had been analyzed yielded fluorescence autocorrelation profiles that were distinct from the monomeric controls POPS and GFP controls (Sarkar-Banerjee et al., 2017). The FCS data for the KRAS4B samples required a 3-component diffusion model for fitting, whereas all of the data for the controls could be fit to a bi-component diffusion model (Sarkar-Banerjee et al., 2017). The majority of cells expressing K101C/E107C gave rise to atypical fluorescence autocorrelation profiles compared with only about half of those expressing K101E (Sarkar-Banerjee et al., 2017). This suggested that the two mutants differ in their ability to form slowly diffusing species, which is consistent with the FRAP data. Further analysis with Raster image correlation spectroscopy (RICS) showed that K101E diffused at a rate similar to POPS while WT and especially K101C/E107C were significantly slower (Sarkar-Banerjee et al., 2017). Number and brightness (N&B) analysis of the RICS images further showed GFP-KRAS4BG12V existed as a combinations of monomers, dimers and larger oligomers (Sarkar-Banerjee et al., 2017). The monomer fraction of GFP-KRAS4BG12V was found to be 38%, which was comparable to the monomer fraction estimated by EM-nanoclustering analysis (∼40%) (Plowman et al., 2005). In this analysis, GFP-KRAS4BG12V was found to exist mostly as a dimer (51%), with a minor percentage of trimer (10%). K101E was predominantly monomeric (73%) with a smaller (23%) fraction of dimers , whereas K101C/E107C was enriched in dimer (58%) and trimer (38%) but was depleted of monomers (Sarkar-Banerjee et al., 2017). Similar results were obtained when ion pairs E98-K165 and D105-K172 were predicted to stabilize larger oligomers including pentamers. For example, double charge-reversion (E98K/D105K) reduced clustering by about 40% without affecting membrane retention, whereas swapping charges had no effect (Sarkar-Banerjee et al., 2017). It has been proposed that KRAS4B self-assembly into oligomers of diverse sizes and shapes involved the use of varying pairwise interactions of i1 and i2 (Sarkar-Banerjee et al., 2017). The resulting structural models explained a number of previous observations (Plowman et al., 2005; Hancock, 2006; Kholodenko et al., 2010), including the average number of proteins per cluster and the average radius of RAS nanoclusters estimated by EM after accounting for the sizes of GFP, antibody, gold nanoparticle and nanocluster geometry (Plowman et al., 2005; Hancock, 2006; Zhou and Hancock, 2015).

### Targeting RAS Nanoclusters for Treating RAS Pathology

As RAS nanoclusters are the main sites for the recruitment and activation of effectors, agents that perturb the RAS nanodomain structure or dynamics should have a therapeutic value against oncogenic RAS. Because PS is a major structural component of KRAS4B nanoclusters, perturbing the PS content of the nanoclusters is a particularly appealing therapeutic avenue. PS is actively transported intracellularly between the endoplasmic reticulum (ER), recycling endosomes, and the PM (Chandra et al., 2012; Schmick et al., 2014; Schmick et al., 2015). Perturbing PS transport can deplete the PS content of the PM and consequentially attenuate the oncogenic activities of mutant KRAS4B. Indeed, treatment of cells by fendiline, an acid sphingomyelinase (ASM) inhibitor (Gulbins et al., 2013), effectively depleted PS in the PM inner leaflet and thereby mislocalized oncogenic mutant KRAS4B from the PM and disrupted its nanoclustering and signaling (van der Hoeven et al., 2013; Cho et al., 2015; van der Hoeven & et al., 2017). ASM converts sphingomyelin (SM) to ceramide (Cer) (Santana et al., 1996). The SM/Cer equilibrium contributes to the vesicular trafficking between the PM and the recycling endosomes that are highly enriched with PS (Chatterjee et al., 2001). The fendiline-disrupted spatiotemporal organization and signaling of oncogenic mutant KRAS4B were selectively restored by the acute addback of natural extracts of PS, but not the natural extracts of other lipids tested including PIP_2_, PC or phosphatidylethanolamine (PE) (Cho et al., 2015). Effects of fendiline on the MAPK-regulated cell proliferation were more pronounced on the oncogenic mutant KRAS4B-transformed tumor cells, but not tumor cells that were independent of oncogenic KRAS4B activities (Cho et al., 2015; van der Hoeven et al., 2013; van der Hoeven & et al., 2017). Fendiline treatment also effectively reduced the sizes of tumors in xenografts composed of tumor cells transformed by mutant KRAS4B, but not those independent of mutant KRAS4B (van der Hoeven & et al., 2017). Taken together, by disrupting PS trafficking from recycling endosomes to the PM, fendiline effectively depletes the PS content in the PM and compromises the spatiotemporal organization, signaling and oncogenic activities of mutant KRAS4B.

Proper intracellular transport of PS can also be blocked or attenuated by staurosporine, an alkaloid isolated from bacterium *Streptomyces* staurosporeus, and analogs. These small molecules include 7-oxostaurosporine (OSS), UCN-01 and UCN-02. Treatment of cells by staurosporines effectively mislocalized PS from the PM to endosomes (Cho et al., 2012). As a result, these staurosporine analogs effectively mislocalized mutant KRAS4B from the PM and disrupted the nanoclustering of KRAS4B left on the PM, which in turn inhibited the mutant KRAS4B-dependent MAPK signaling (Cho et al., 2012). Additional strategies for interfering with the PS transport involve perturbing the exchange of PS between the PM and the endoplasmic reticulum (ER), via altering the expression of oxysterol-related binding proteins, ORP5 and ORP8. ORP5 and ORP8 regulate the exchange of phosphoinositol 4-monophosphate (PI_4_P) in the PM and PS in the ER (Moser von Filseck et al., 2015; Moser von Filseck and et al., 2015; Sohn et al., 2016). Concordantly, treatment by a selective inhibitor of PI4-kinase IIIα (PI4KIIIα) that converts phosphoinositol (PI) to PI_4_P (Waring et al., 2014; Boura and Nencka, 2015; Raubo et al., 2015), called compound 7, depleted the PS levels in the PM by reducing the PI_4_P/PS exchange (Kattan et al., 2019). Indeed, Compound 7 effectively mislocalized oncogenic mutant KRAS4B from the PM and disrupted the nanoclustering of mutant KRAS4B (Kattan and et al., 2019). Compound 7 also selectively compromised the proliferation of human tumor cell lines transformed by mutant KRAS4B, but not those independent of KRAS4B (Kattan and et al., 2019). Taken together, pharmacologically targeting the PS transport between endomembranes and the PM effectively and selectively perturbs the oncogenic activities of mutant KRAS4B.

As described above, phosphorylation of Ser181 mislocalizes KRAS4B from the PM and decreasing its clustering on the PM (Bivona et al., 2006; Cho et al., 2016). This is correlated with the switch of lipid sorting preference from PS to the relatively less abundant anionic phospholipid PIP_2_ (Zhou et al., 2017). Protein kinase C (PKC) and protein kinase G (PKG) directly phosphorylate KRAS4B at Ser181, resulting in changes in the spatiotemporal organization of oncogenic mutant KRAS4B and inhibition of mutant KRAS4B-dependent MAPK signaling (Bivona et al., 2006; Cho et al., 2016). Several groups of compounds have been shown to promote the phosphorylation of Ser181 of KRAS4B and perturb oncogenic KRAS4B activities. Specifically, the PKC activator, bryostatin-1, mislocalized oncogenic mutant KRAS4B from the PM and induced apoptosis (Bivona et al., 2006). Additionally, a number of small molecules have been shown to activate the AMP-activated protein kinase (AMPK) → eNOS → soluble guanylyl cyclase (sGC) → cyclic GMP (cGMP) → PKG cascade and promote the phosphorylation of Serine 181 of KRAS4B (Cho et al., 2016). These PKG-activating molecules include AMPK activators oligomycin A, neoantimycin, antidiabetic drug metformin and aminoimidazole-4-carboxamide riboside (AICAR) (Cho et al., 2016). Nitric oxide donor, diethylamine nitric oxide (DEA-NO), promotes the generation of sGC in the production of cGMP, the main substrate of PKG. Sildenafil inhibits PDE5 hydrolyze cGMP and lead to the further accumulation of cGMP (Cho et al., 2016). These PKG activators attenuated the PM localization and nanoclustering of oncogenic mutant KRAS4B on the PM, and inhibited the mutant KRAS4B-dependent MAPK signaling (Cho et al., 2016). Thus, altering the selective lipid sorting of KRAS4B by inducing phosphorylation of Serine 181 effectively attenuates the oncogenic activities of mutant KRAS4B.

Monobodies and ankyrin repeat proteins (DARPins) have also been utilized to directly target KRAS4B dimers. Specifically, integration of NMR spectroscopy, X-ray diffraction of crystal structures, fluorescence imaging of intact cells, EM-spatial analysis, as well as signaling and functional assays have revealed that a monobody called NS1 bound to the α4, β5 and α5 interface of HRAS and KRAS and disrupted their dimerization and nanoclustering. As a result, NS1 perturbed effector binding, inhibited MAPK signaling and cell proliferation regulated by oncogenic mutants of HRAS and KRAS (Spencer-Smith et al., 2017). Similarly, several DARPins have been shown to bind the i1 dimer interface involving helices α3, α4 and loop 7 or the switch 1 region and inhibited KRAS signaling and RAS-dependent proliferation (Guillard et al., 2017; Bery et al., 2019). Taken together, existing data suggest that directly targeting dimer interfaces of RAS is also an effective strategy for compromising the oncogenic activities of RAS.

## Conclusion

**FIGURE 5:**
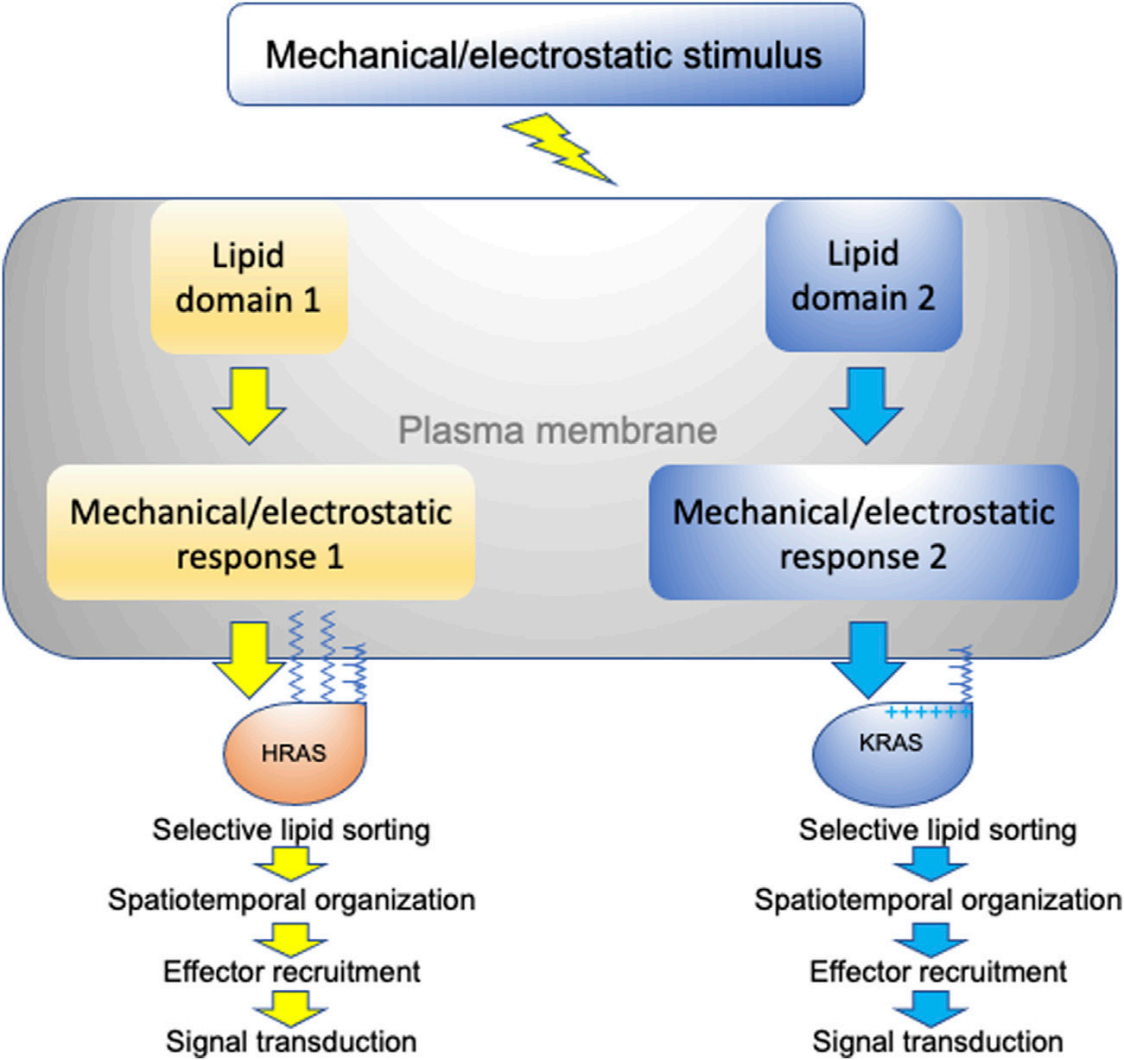
A schematic description of RAS nanoclusters acting as transition hubs to couple extracellular stimuli with intracellular signaling networks. In a highly heterogeneous plasma membrane, different proteolipid nanodomains possess distinct biophysical properties and respond to membrane perturbations in distinct manners. Diverse changes in lipid packing and lateral diffusion of plasma membrane domains alter the spatiotemporal organization of RAS isoforms, which in turn perturb effector recruitment and signal intracellular transmission.

We have discussed how different RAS isoforms undergo spatial segregation on the plasma membrane for efficient signal transduction and function. More specifically, we have focused on the intricate capabilities of RAS proteins to selectively sort lipids in a headgroup- and acyl chain structure-dependent manner. This specific lipid sorting capability not only allows RAS proteins to recruit effectors in an isoform-specific manner, but also allows RAS nanoclusters to sense and respond to various membrane perturbations in distinct manners (summarized in Figure 5). This is because plasma membrane domains that vary in lipid and protein content as well as mechanical and electrostatic properties respond to membrane perturbations in distinct manners. We therefore propose that RAS/lipid nanoclusters act as important transition hubs on the cell surface, where extracellular mechanical and electrostatic stimuli are relayed to distinct intracellular signal output. These nanometer-sized transition hubs intricately connect extracellular stimuli with intracellular signaling networks and may contribute to mechanosensing and mechanotransduction.
